# Metabolic Controls on Epigenetic Reprogramming in Regulatory T Cells

**DOI:** 10.3389/fimmu.2021.728783

**Published:** 2021-08-05

**Authors:** Jingli Lu, Yan Liang, Haiyang Meng, Ailing Zhang, Junjie Zhao, Chengliang Zhang

**Affiliations:** ^1^Department of Pharmacy, The First Affiliated Hospital of Zhengzhou University, Zhengzhou, China; ^2^Henan Engineering Research Center of Clinical Mass Spectrometry for Precision Medicine, Zhengzhou, China; ^3^Zhengzhou Key Laboratory of Clinical Mass Spectrometry, The First Affiliated Hospital of Zhengzhou University, Zhengzhou, China; ^4^Department of Pharmacy, Tongji Hospital, Tongji Medical College, Huazhong University of Science and Technology, Wuhan, China

**Keywords:** regulatory T cells, metabolism, epigenetics, immune suppression, metabolites

## Abstract

Forkhead box protein 3 (Foxp3^+^)-expressing regulatory T (Treg) cells are a unique CD4^+^T cell subset that suppresses excessive immune responses. The epigenetic plasticity and metabolic traits of Treg cells are crucial for the acquisition of their phenotypic and functional characteristics. Therefore, alterations to the epigenetics and metabolism affect Treg cell development and function. Recent evidence reveals that altering the metabolic pathways and generation of metabolites can regulate the epigenetics of Treg cells. Specifically, some intermediates of cell metabolism can directly act as substrates or cofactors of epigenetic-modifying enzymes. Here, we describe the metabolic and epigenetic features during Treg cell development, and discuss how metabolites can contribute to epigenetic alterations of Treg cells, which affects Treg cell activation, differentiation, and function.

## Introduction

Forkhead box protein 3 (Foxp3^+^)-expressing regulatory T (Treg) cells are a subset of CD4^+^ T cells that are essential for maintaining immune tolerance (1). In non-lymphoid tissues, they can also modulate non-immunological processes, such as wound healing and tissue repair (2). To acquire their phenotypic and functional hallmarks, Treg cells must generate a specific epigenetic signature (3) and rely on their unique metabolic requirements (4). The alteration of either of these features can lead to Treg cell instability and functional disruption. Increasingly, research has focused on the interplay between epigenetic and metabolic features, with the recognition that cellular metabolism can regulate epigenetic states when the intermediary metabolism generates substrates or cofactors for chromatin regulation (5, 6). Although integrated analysis of the complex interactions between epigenetics and the cellular metabolism that reprograms Treg cells is a relatively new area, such a conceptual understanding will be important for the design of effective strategies aimed at manipulating Treg cells in cancer and autoimmune diseases.

It is becoming increasingly clear that Treg cells have their own metabolic preferences at different stages of activation to support their energetic and biosynthetic demands (4). Importantly, some intermediates can also regulate the epigenetics of Treg cells and, as a consequence, influence cell differentiation and function (7). There are several universal principles regarding the role of the metabolism-epigenetics axis that facilitate epigenetic dynamics under metabolic changes (8). For example, S-adenosylmethionine (SAM) is the methyl donor for DNA and histone methylation, acetyl-coenzyme A (acetyl-CoA) is the acetyl donor for histone acetylation; tricarboxylic acid (TCA) metabolites related to α-ketoglutarate (α-KG) are important for demethylases, and NAD^+^ availability regulates the function of the sirtuin (SIRT) family of enzymes (9). Thus, it is not difficult to speculate that fluctuations in metabolite levels could modulate the activities of epigenetic enzymes and therefore influence the epigenetic state during Treg cell development.

From this perspective, we summarize the current knowledge of metabolic reprogramming and epigenetic features in Treg cells within different contexts. We then discuss how metabolism controls epigenetic modification and evaluate the functional molecular consequences of these modifications for Treg cell activation, differentiation, and function.

## An Overview of Metabolic Reprogramming in Treg Cells

**Figure 1:**
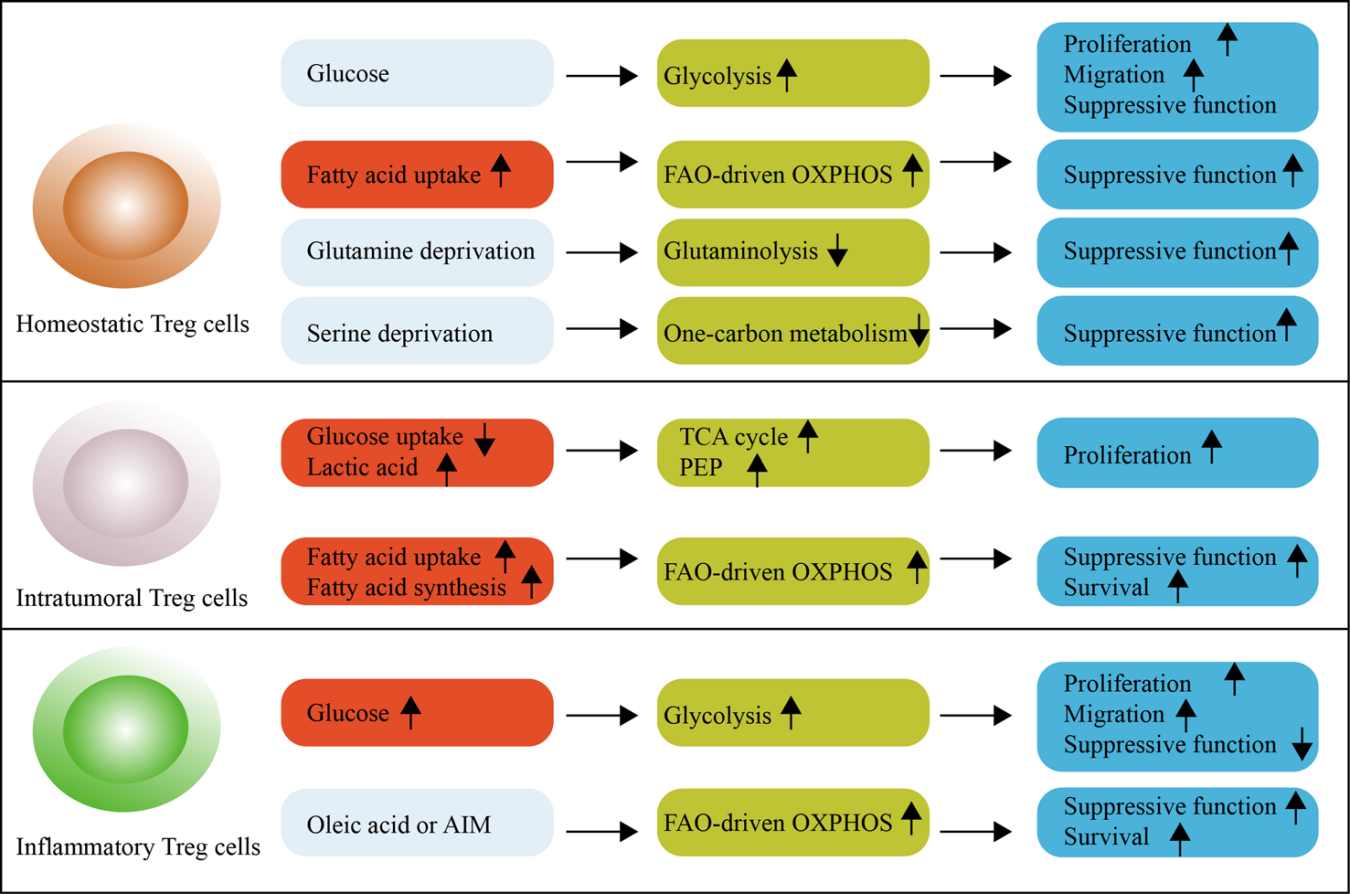
An overview of metabolic reprogramming in Treg cells. Glycolysis is engaged to fuel the proliferation and migration of Treg cells, but is associated with reduced suppressive function and long-term instability in mice. Glycolysis is required for the proliferation, differentiation and suppressive function of human Treg cells. Treg cells increase the reliance on OXPHOS and FAO. Under homeostatic conditions, Treg cells readily take up exogenous fatty acids for this purpose. Serine-driven one-carbon metabolism and glutamine-driven glutaminolysis are not necessary for Treg cells because their absence promotes the differentiation and function of Treg cells. Intratumoral Treg cells use lactic acid to feed the TCA cycle and generate PEP to fuel proliferation. These cells reprogram lipid metabolism by upregulating lipid uptake and *de novo* lipid synthesis to support FAO-driven OXPHOS metabolism. In autoimmune diseases, inflammatory Treg cells exhibit a dysfunctional suppressive function, which can be supported by high levels of glycolytic metabolism. Oleic acid counteracts this effect. The OXPHOS of lipids can also be promoted by the DNA-binding inflammasome receptor AIM. FAO, fatty acid oxidation; OXPHOS, oxidative phosphorylation; PEP, phosphoenolpyruvate; TCA cycle, tricarboxylic acid cycle.

Treg cells require energy for survival and function; nutrient processing through distinct metabolic pathways produces adenosine triphosphate (ATP) to meet these energy requirements (4). The metabolic pathways of Treg cells are affected by the availability of nutrients such as glucose, fatty acids, and amino acids (**Figure 1**).

Glucose is involved in both glycolysis and oxidative phosphorylation (OXPHOS). Glycolysis is a relatively inefficient pathway for the generation of cellular ATP (only two molecules); however, it can be rapidly activated *via* the induction of enzymes involved in this pathway (10). Potentially more important than rapid ATP generation, however, is the ability of glycolysis to produce various intermediates to support anabolic reactions in cells (11). For these reasons, although mouse Treg cells differentiated from naive T cells is not characterized by increased glycolysis (12, 13), glycolysis is observed in proliferating, migrating and effector Treg cells (14, 15). Proliferative Treg cells activate mTORC1 and glucose uptake (16), but high-glucose conditions impair suppressive function and long-term stability *in vitro* (17). Surprisingly, unlike mouse Treg cells, the requirement of glycolysis seem to differ in human Treg cells (18, 19). Freshly-isolated human Treg cells are glycolytic, and glycolysis is necessary for the proliferation, differentiation and suppressive function *in vitro* (18, 19). Consequently, inhibition of glycolysis impairs the generation and functions of human Treg cells, accompanied with reduced the expression of Foxp3 and other Treg cell markers (18, 19).

Furthermore, glucose metabolism of Treg cells differs in the tumor microenvironment and autoimmune diseases. Indeed, glucose uptake is upregulated in dysfunctional Treg cells from autoimmune diseases (20), but is notably low in intratumoral Treg cells (17). Studies in mice have revealed that lower glucose uptake is a universal phenotype of intratumoral Treg cells (17, 20). Intratumoral Treg cell avoidance of glucose metabolism is functionally important and may be mediated by CTLA-4 overexpression (21). By blocking CD28 signaling, decreased glucose utilization can ensure the functional stability of Treg cells (21). Intratumoral Treg cells then increase their uptake of the glycolytic by-product, lactic acid (17). Treg cells use lactic acid, not only to feed the TCA cycle, but also to generate phosphoenolpyruvate (PEP), which is essential for fueling the proliferation of Treg cells within tumors (17). Consequently, treatment with lactate maintains the suppressive function of Treg cells against the negative effects of high-glucose conditions (17). Metabolic support by lactic acid reflects the metabolic flexibility of using a carbon source in intratumoral Treg cells according to the nutrient milieu. Thus, Treg cells display broad heterogeneity in their metabolism of glucose within context-specific tissues and diseases. As such, increased glucose uptake is considered a hallmark functional change in Treg cells.

Lipid metabolism is important for Treg cell development (22). It is now accepted that the fatty acid oxidation (FAO)-driven OXPHOS metabolic reprogramming maintains its suppressive phenotype, which is further promoted by the expression of Foxp3 (13, 16, 23). Intriguing to know that fatty acids produced by gut microbiota and the composition of gut bile acid metabolites mediate enhancement of Treg cell differentiation and cell homeostasis (24–26). Under homeostatic conditions, mouse Treg cells do not depend on *de novo* fatty acid synthesis, but readily take up exogenous fatty acids for this purpose (27). Thus, inhibiting acetyl-CoA carboxylase 1 (ACC1), an enzyme that initiates the generation of long-chain fatty acids, does not affect Treg cell differentiation and function (27). In contrast, intratumoral Treg cells rely on both exogenous fatty acids and *de novo* fatty acids (14, 20, 28). Specially, intratumoral Treg cells are highly expressed fatty acid transporters CD36 (14, 28), which enhance lipid uptake and activate PPAR-β pathways to support intratumoral Treg cell survival and suppressive functions (28). Intratumoral Treg cells also actively rewire transcription factor SREBP-dependent *de novo* lipid biosynthesis, contributing to the TCR-induced functional maturation and induction of PD-1 expression (20). In the case of autoimmune disease, the OXPHOS of lipids is promoted by the DNA-binding inflammasome receptor AIM, which attenuates Akt phosphorylation, mTOR and Myc signaling (29). Interestingly, more recent work using tissue-resident Treg cells from patients with multiple sclerosis reveals that oleic acid is necessary to counteract the negative effects of upregulated glucose uptake (30). Oleic acid amplifies FAO-driven OXPHOS metabolism, creating a positive feedback mechanism that increases the expression of Foxp3 and the phosphorylation of STAT5, thereby enhancing suppressive function (30). It is now clear that context-dependent lipid metabolic adaption engaged by Treg cells orchestrates signaling pathways to support suppressive activity.

Amino acid metabolism supports protein and nucleotide synthesis needed for rapid cell growth (31). As such, subunits of amino acid transporters, including SLC7A5, SLC43A2, SLC7A1, especially SLC3A2 and SLC7A11, have been found highly upregulated during Treg cell proliferation and activation in human and mouse studies (32–34). Branched-chain amino acids, including isoleucine, are required for *in vivo* maintenance of the proliferative state of mouse Treg cells, which are reliant on the amino acid transporter SLC3A2-dependent metabolic reprogramming (33). In TCR-stimulated human Treg cells, cystine/glutamate antiporter SLC7A11 acts as a key molecular determinant in the control of Treg cell proliferation in normal and pathological conditions (34). Consistent with these observations, arginine and leucine are required to license Treg cells’ response to TCR stimulation (32); whereas Treg cells from mice fed with reduced isoleucine, leucine, or valine have decreased the proliferation and suppressive ability (33). In addition, amino acid metabolic enzymes and intermediates are also an important factor in determining Treg cell induction. For example, the activity of the amino acid-consuming enzyme indoleamine 2,3-dioxygenase (IDO) can strongly promote Treg cell differentiation *in vitro* (35). Tryptophan metabolites, especially kynurenine, which is generated through IDO, can bind the aryl hydrocarbon receptor on T cells and promote Treg cell induction (36). However, there is also evidence revealing that SLC1A5 and SLC7A5, which are responsible for the uptake of glutamine and leucine, may not be necessary to generate Treg cells (37, 38). Interestingly, in the setting of T cell differentiation, glutamine deprivation even promotes the generation and function of Treg cells while inhibiting Th1 cell (39, 40); Limitation of serine availability preserves Foxp3 expression and Treg cell function (41). In this regard, such reliance on different amino acids allows the opportunity for metabolic selection in Treg cell development.

Together, Treg cells adopt a coordinated metabolic profile with engagement of glycolysis, FAO-driven OXPHOS and amino acid metabolism, which are not mutually exclusive during Treg cell development. In addition, the specific metabolic reprogramming of Treg cells determine their differentiation and function in different contexts, with Treg cells able to alter the metabolic phenotype to adapt to the environment, especially in non-lymphoid tissues.

## Epigenetic Landscape of Treg Cells

The Treg cell-specific epigenetic landscape begins to be established in early stages of thymic Treg cell generation before the expression of Foxp3 and other Treg cell signature genes (42, 43). In non-lymphoid tissues, Treg cells show a stepwise acquisition of chromatin accessibility and reprogramming toward the non-lymphoid tissue Treg cell phenotype (44). Treg cells from different non-lymphoid tissues have a distinct chromatin accessibility profiling (44), but exhibit a conserved tissue-repair chromatin signature both in human and mice (45). As we discuss here, epigenetic regulation in Treg cells mainly includes DNA methylation, histone methylation and acetylation, which influences gene expression patterns in a coordinated manner.

DNA methylation is the most important Treg-specific epigenetic signature (42, 46–49), the process by which a methyl (-CH3) group is added to the ϵ-amino group of amino acid residues on DNA (50). DNA methylation is generally associated with transcriptional repression; therefore, comparison of genome-wide DNA methylation profiles between mouse Treg and conventional T cells reveal that naturally occurring Treg cells (nTreg cells) frequently display hypomethylation at Treg cell-associated gene loci (such as *Foxp3*, *Ctla4*, *Tnfrsf18*, and *Ikzf2*), which contributes to Treg cell suppressive activity and lineage stability (47). Notably, much of our current understanding of the role of methylation in Treg cells comes from a particular region at the *Foxp3* locus; specifically, the distal enhancer elements known as conserved non-coding sequences (CNSs) (51, 52). Demethylation of CNS2 in the *Foxp3* gene enable the binding of transcription factors such as RUNX1–CBFβ, and Foxp3 itself (53). Foxp3 cannot blind to fully methylated CNS2 *in vitro* (53). To support this, Treg cells generated *in vitro* (iTreg cells) with unstable Foxp3 expression possess a methylated or partially demethylated CNS2 region (47, 51, 53, 54), while Treg cells with stable Treg cell-specific DNA hypomethylation allow them to transfer *in vivo* and effectively suppress the immune response (55). However, inflammatory gene loci (such as *Tbx1*) appears to have methyl-DNA marks mediated by the epigenetic regulator Uhrf1, which represents a stable Treg cell identitiy by repressing effector T cell transcriptional programs (56, 57). In this way, DNA methylation regulation appears to be flexible, allowing for an open state at genes required for Treg cell differentiation and function while maintaining methyl-DNA marks at the inflammatory gene locus.

Unlike DNA methylation, histone methylation either activates or represses gene expression depending on which residue is modified and the number of methyl groups incorporated (58). Global mapping reveals that nTreg cells have the largest unique H3K4me3 and H3k27me3 islands, compared to conventional T cells (59). Yet, enhanced H3K4me3 modification in the Treg cell-associated genes is also detected in iTreg and conventional T cells (47). It suggests that the histone methylation may be not specific for the nTreg cell lineage. However, when human Treg cells lose their Foxp3 signature, they exhibit decreased abundance of permissive H3K4me3 within the downregulated Treg cell signature genes (such as *Foxp3* and *Ctla4*), and increased abundance of H3K4me3 within the Th2-associated genes (such as *Il-4* and *Il-5*); the H3K27me3 profile, a repression-related histone modification, does not change significantly (60). Consistent with this, H3K4me3 modification, but not H3K27me3, is found to accumulate in the majority of promoters of transcriptional start site (TSS) clusters (61). Of note, Foxp3-bound sites in activated Treg cells are specifically enriched for H3K27me3, which are required for Foxp3-mediated repressive chromatin under inflammatory conditions (62). Adding complexity, Foxp3 itself at Treg cell specific-super enhancers (SEs) region shows a stronger H3K4me1, and weaker H3K27me3 (42). Thus, further work is needed to determine what role of specific histone methylation at specific locus may have during Treg cell activation and development.

Histone acetylation is another important chromatin modification in Treg cells, which acts on targeted regions of chromatin to regulate specific gene transcription, or acts in a more global manner (63). Acetylation neutralizes the positive charge of lysine, leading to a more open chromatin configuration that enables DNA binding, whereas histone deacetylation is typically associated with condensed chromatin and transcriptional repression (6). The importance of histone acetylation in the context of Treg cells is exemplified by the effects of pan-histone deacetylase (HDAC) inhibition in mice, which increases acetylated histone 3 and boosts thymic production of Treg cells with enhanced suppression (64), highlighting the relevance of the overall acetylation status for controlling the generation and functional responses of Treg cells. Accordingly, Treg cell-specific hypomethylation is accompanied by histone acetylation and an open chromatin status to regulate the expression of Treg cell genes, which mediates important influences on the susceptibility to autoimmune disorders (65). Compared with conventional T cells, H3K27ac deposition at the *Foxp3* promoter occur exclusively in Treg cells (66); conserved Foxp3 binding is associated with decreased H3K27ac in both human and mouse Treg cells (67). These data suggest that H3K27ac modification in specific locus is important for Treg cell differentiation and function.

The recent technique of assay for transposase-accessible chromatin with high-throughput sequencing (ATAC-seq) has enabled the genome-wide identification of open chromatin regions (68). Global changes in chromatin accessibility identify 3833 loci with sex-dependent differential accessibility in visceral adipose tissue (VAT) Treg cells (69). These sites include male VAT Treg cell signature such as *Il1rl1*, *Il10*, *Pparg* and *Klrgl*, which shows increased accessibility (69). The application of single cell ATAC-seq allows for investigation of heterogeneity in non-lymphoid tissue Treg cells. For instance, single-cell ATAC-seq identifies tissue-repair Treg cell chromatin landscape, and this signature is likely induced by the transcription factor BATF which is critical for tissue Treg cell differentiation, recruitment, and maintenance (45, 70).

Collectively, these data indicate that epigenetics are important for multiple aspects of Treg cell reprogramming. However, the mechanism of Treg cell functional coordination through regulation of the epigenetic landscape in the context of the promoter, enhancer, and gene body of a specific gene requires further investigation. Importantly, these data also suggest that manipulation of the epigenetic landscape could provide an important strategy for controlling Treg cells in a context-dependent manner.

## Metabolic Control of Epigenetics in Treg Cells

Immune cell metabolism studies have focused on understanding how metabolites modulate their plasticity by affecting epigenetic reprogramming, as well as how the interplay between metabolic pathways and epigenetic modification support their activation, differentiation, and function. However, this is a relatively new area of Treg cell biology. Thus, unraveling how metabolism and epigenetics coordinate with each other to regulate cell plasticity and/or function will be a key area of research for Treg cells.

**Figure 2:**
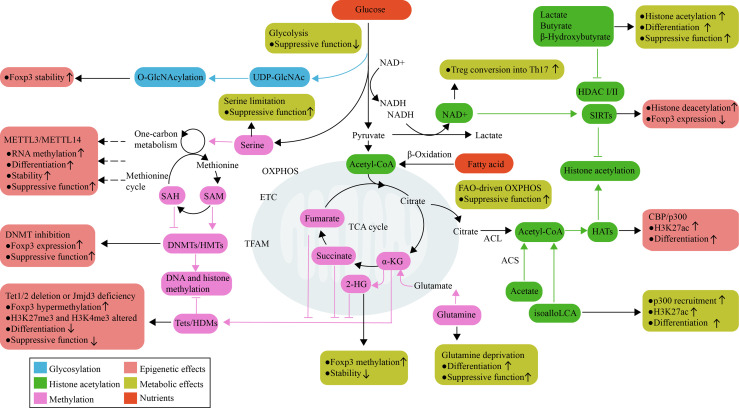
Metabolic control of epigenetics in Treg cells. Treg cell activation, differentiation, and function are linked to metabolic reprogramming. Metabolic pathways not only process nutrients to produce ATP and meet energy requirements, but also use available metabolites as subtracts and cofactors for epigenetic enzymes that control Treg cell development. Glucose and glutamine are used to fuel O-GlcNAcylation, which can stabilize Foxp3. The mitochondria-derived metabolites α-KG, 2-HG, succinate, and fumarate are important for the function of DNA and histone demethylases; their effects oppose the actions of HMTs and DNMTs, which are regulated by amino acids that initiate one-carbon metabolism pathways and the methionine cycle. Histone acetylation is dependent on the supply of acetyl-CoA, which can be generated through a range of metabolic pathways. The product of bacterial anaerobic fermentation butyrate, as an inhibitor of HDACs, increases histone H3 acetylation in the *Foxp3* locus and promotes Treg cell function. The SIRT family of enzymes promotes deacetylation function, which is dependent on NAD^+^ availability as a cofactor and regulated by the NAD^+^/NADH ratio. RNA methylation also requires the transfer of a methyl group; however, the contribution of metabolites to RNA methylation has not yet been explored in Treg cells. 2-HG, 2-hydroxyglutarate; α-KG, α-ketoglutarate; ACL, ATP-citrate lyase; ACS, acetyl-CoA synthetase; DNMTs, DNA methyltransferases; ETC, electron transport chain; GlcNAc, N-acetylglucosamine; HATs, Histone acetyltransferase; HDMs, histone demethylases; HMTs, histone methyltransferases; OXPHOS, oxidative phosphorylation; SAH, S-adenosylhomocysteine; SAM, S-adenosylmethionine; SCFAs, short-chain fatty acids; SIRT, sirtuin; TCA, tricarboxylic acid; Tets, ten-eleven translocation family members.

In this section, we focus on the role of specific metabolites that reprogram the transcriptional profile of Treg cells through epigenetic changes, as achieved by changes in differentiation and function. We also discuss whether the effects of metabolite-conditioned epigenetic modifications are consistent with those of the corresponding metabolic pathway. (**Figure 2**)

### Methyltransferase Regulation in Treg Cells by SAM as a Methyl Donor

DNA and histone methylation require DNA methyltransferases (DNMTs) and histone methyltransferases (HMTs), which add methyl groups to DNA or lysine/arginine residues of histones, respectively. The activities of these enzymes influence the methylation landscape, and therefore activate or repress target gene expression. As demonstrated in Treg cells, the pharmacological inhibition of DNMTs is sufficient to induce Foxp3 expression in mature conventional CD4^+^ T cells and potentiate suppressive function (71–74), suggesting the importance of these enzymes in Treg cells.

Although structurally and functionally diverse, DNMTs and HMTs share a similar reaction mechanism, i.e., the transfer of a methyl group. SAM is one of the most thoroughly-described methyl donors for methylation, which is generated in the one-carbon metabolism pathway that encompasses both the folate and methionine cycle (75). Several amino acids, such as threonine, serine, and glycine, can initiate the one-carbon metabolism pathways in the folate cycle, thereby promoting the production of SAM in the methionine cycle (76, 77). The downstream metabolite of SAM is S-adenosylhomocysteine (SAH), which competitively inhibits DNMTs and HMTs (75). In a variety of cellular systems, it has been proven that alterations in the intracellular concentrations of SAM and SAH determine the amount of DNA and histone methylation, thereby altering gene transcription (78, 79). Given the complexity of SAM and SAH metabolism, it implies that multiple metabolic inputs are closely linked to methylation levels.

From a metabolic perspective, recycling homocysteine to methionine induced by 1,25-Dihydroxyvitamin D3 is associated with CD4^+^T cell DNA methylation and Treg cell stability, which reverses autoimmune neurodegenerative disease in mice (80). One-carbon units fed by serine metabolism are synergistically integrated into the methionine cycle to fuel the generation of SAM (75), which may increase histone and DNA methylation; however, this needs to be formally demonstrated in Treg cells. In this regard, increased serine metabolism can enhance Treg cell proliferation but downregulate Foxp3 expression, whereas restriction of serine availability by glutathione is required for the suppressive function of Treg cells (41). In addition, *all-trans* retinoic acid (atRA), the major vitamin A metabolite, also increases histone acetylation on *Foxp3* gene promoter and CpG demethylation in the region of *Foxp3* locus, stabilizing human nTreg cells under inflammatory conditions (81, 82). Although little is known about how metabolites synergistically orchestrate SAM availability in Treg cells, there is no doubt that additional connections will unfold after further investigation of metabolites and the metabolic pathways that may modify methyltransferases.

### Demethylase Regulation in Treg Cells by α-KG, 2-Hydroxyglutarate (2-HG), Succinate, and Fumarate

Active removal of histone and DNA methylation is mediated by the Tet family of DNA demethylases (ten-eleven translocation family members, Tets) and histone demethylases (HDMs). Tet1/2 catalyzes the conversion of 5-methylcytosine (5mC) to 5-hydroxymethylcytosine (5hmC) in Foxp3 to establish a Treg cell-specific hypomethylation pattern and stable Foxp3 expression in mouse lymphoid tissues (83). In contrast, Tet1/2 deletion leads to Foxp3 hypermethylation, impairs Treg cell differentiation and function (83). With respect to HDMs, deficiency in Jmjd3 alters H3K27me3 and H3K4me3 levels, which inhibits mouse Treg cell differentiation (84). As methylation levels are responsive to enzyme activity, the regulation of these processes will undoubtedly become an important field in Treg cell research.

From a metabolic perspective, the demethylation mechanism is associated with α-KG, 2-HG, succinate, and fumarate, which are key metabolites of the TCA cycle. Tets and HDMs belong to the class of α-KG-dependent dioxygenases that use α-KG and oxygen as substrates (85). Succinate, fumarate and 2-HG, which are metabolism-derived structural analogs of α-KG, act as competitive inhibitors of these α-KG-dependent dioxygenases (85, 86). These metabolites can be derived from either glucose or glutamine, and participate in both anabolism and catabolism (87). Consequently, metabolic interventions that involve alterations in these metabolites modulate the activity of Tets and HDMs, which in turn regulate Treg cell activation, differentiation, and function. For example, the deamination of glutamate to form α-KG is the last step in glutamine catabolism, which allows glutamine to fuel the TCA cycle, and is therefore crucial for decreasing Foxp3 expression and inhibiting suppressive function in Treg cells (39, 40). In addition, glutamine catabolism maintains a high level of intracellular α-KG and a high intracellular α-KG/succinate ratio, which is sufficient for regulating multiple chromatin modifications, including H3K27me3 and Tet-dependent DNA demethylation (88).

The effect of the glutamate-dependent metabolic pathway on the development of Treg cells through epigenetic modification has been directly validated in mice (89). Inhibition of the conversion of glutamate to α-KG prevents the production of 2-HG, reduces methylation of the *Foxp3* gene locus, and increases Foxp3 expression (89). This is because 2-HG markedly increases the methylation levels of the *Foxp3* promoter in differentiating Treg cells (89). In line with this, the knockdown of isocitrate dehydrogenase (IDH) 1 and IDH2, which catalyzes the reduction of α-KG to 2-HG by NADPH, reduces methylation levels at the *Foxp3* promoter and CNS2 regions (89). Intriguingly, increased conversion of glutamate to α-KG leads to much greater accumulation of 2-HG in Th17 cells than in Treg cells, which correlates with hypermethylation of the *Foxp3* gene locus and reduces the mRNA and protein levels of Foxp3 in fully differentiated Th17 cells (89). These results suggest that different cell types may exhibit differential sensitivity to 2-HG levels; thus, manipulating a single step in a glutamate metabolic pathway could regulate the Th17/Treg balance by affecting the methylation state of *Foxp3* (89).

Mitochondrial respiratory capacity is critical for the engagement of metabolites in the mitochondrial TCA cycle; mitochondrial perturbation also contributes to the changes of metabolites (e.g., α-KG, succinate, and fumarate), which leads to alterations in epigenetic modifications depending on cell type and conditions (87). Mitochondrial Transcription Factor A (Tfam) is essential for mitochondrial respiration and controls transcription and replication of mitochondrial genome (90). Ablation of Tfam impairs Treg cell maintenance in non-lymphoid tissues and tumor microenvironment, but does not affect Treg cells in the steady state in lymphoid organs (91). Mechanistically, Tfam-deficient Treg cell switch OXPHOS toward glycolysis, a metabolic pathway that impairs the function and stability of Treg cells (91). Consistently, Tfam-deficient Treg cells exhibit increased DNA methylation, specifically at the Treg-specific demethylation region of the *Foxp3* locus (91). Of note, it is unclear which mitochondrial metabolism metabolites influence DNA methylation in Treg cells during mitochondrial perturbation induced by Tfam deficiency. However, in erythroid cells, Tfam deletion results in aberrant histone acetylation and an increase in the abundance of the metabolite β-hydroxybutyrate, which is known to inhibit histone deacetylases (92). These data support cell-specific activities of Tfam in regulating epigenetic modifications.

Studies have pinpointed a metabolic-epigenetic role for mitochondrial respiratory chain complex III in mouse Treg cells (93). Loss of complex III in Treg cells results in global DNA hypermethylation without affecting the methylation status of canonical Treg cell genes (93). This effect is dependent on the increase of the metabolites 2-HG and succinate, which inhibits the Tet family of DNA demethylases (93). Although mice lacking the mitochondrial complex III in Treg cells do not alter Treg cell proliferation and survival, Treg cells display a loss of suppression capacity, which leads to the development of fatal inflammatory disease early in life (93). These data point to the crucial role of mitochondrial function in the regulation of Treg cell development *via* modification of DNA methylation; however, it is currently unclear why the mitochondrial respiratory capacity is directed to methylation at specific genomic locations under different contexts.

### Histone Acetyltransferase (HAT) Regulation in Treg Cells by acetyl-CoA

Histone acetylation involves the transfer of an acetyl group to the ϵ-amino group of a histone lysine residue, which is catalyzed by multiple families of HATs. In addition to regulating chromatin accessibility by acetylating lysine residues within histone protein, HATs play important roles in regulating the acetylation and function of many non-histone proteins (94). Indeed, in comparison to HAT-mediated Foxp3 acetylation, the role of HATs on histone acetylation in Treg cells is less well understood (95–97). Only one study profiles CBP/p300-meidated H3K27 acetylation, which regulates transcriptional network and drives differentiation of human Treg cells (98). Nevertheless, epigenetic changes modified by HATs can be an important driver of Treg cell function.

HATs-mediated histone acetylation requires the availability of acetyl-CoA, while the product CoA-SH inhibits acetyltransferase activity (99). In addition, other acyl-CoA also influences the activity of HATs. For example, palmitoyl-CoA is a potent inhibitor of HAT activity (100); Crotonyl-CoA, conversely, is used as an alternative substrate for the acetyltransferase p300-catalyzed histone crotonylation (101). It suggests that optimal acetyltransferase activity requires an increased local acetyl-CoA to CoA-SH ratio, and appropriately relative concentration of acyl-CoA and acetyl-CoA, which determines the catalytic activity and substrate specificity of HAT enzymes (102–104). Therefore, metabolic pathways leading to the production or consumption of acetyl-CoA, such as fatty acid oxidation (105) and glucose metabolism (103, 106), are thus able to shape the histone acetylation landscape by modulating the activity of HATs. Several enzymes involved in the production of acetyl-CoA also regulate the deposition of acetylation marks, including acetate-dependent acetyl-CoA synthetase 2 (ACSS2) and citrate-dependent ATP-citrate lyase (ACL) (107). However, the details of these metabolic pathways and the enzymes involved in histone acetylation have not been analyzed in the context of Treg cell development.

The availability of glucose and glycolytic activity influence global levels of histone acetylation through the generation of acetyl-CoA (103, 106). Lactate dehydrogenase A (LDHA), an enzyme that supports aerobic glycolysis in T cells, maintains high levels of acetyl-CoA to enhance histone acetylation (108). Ablation of LDHA diminishes H3K9ac in *Ifng* promoter, but does not affect the number of thymic Treg cells (108). However, as we discussed above, glycolysis itself negatively affects suppressive function of Treg cells. Thus, the glucose-driven generation of acetyl-CoA and histone acetylation fail to explain the observation that glucose uptake is associated with dysfunctional Treg cells.

Indeed, the possible metabolic association between histone acetylation and Treg cell function is that the major source of carbon for histone acetylation is lipid-derived acetyl-CoA (105). By repressing both glucose and glutamine metabolism, fatty acid oxidation reprograms the cellular metabolism, leading to increased lipid-derived acetyl-CoA, which is reflected in increased acetate, citrate, and histone acetylation (105). Thus, these data imply that lipid metabolism on acetyl-CoA may specifically lead to a state of acetylation, which is an important feature of Treg cell stability. However, this hypothesis has not been directly tested in Treg cells, but there is evidence that some metabolites can feed intracellular acetyl-CoA pool to enhance histone acetylation. The bile acid metabolite isoalloLCA increases recruitment of the HAT p300 and H3K27ac levels at the *Foxp3* promoter region, and promotes the differentiation of Treg cells (109). Although lacking the ability to generate Treg cells, acetate has been shown to influence levels of histone acetylation and chromatin accessibility (110). However, further investigation is required to ascertain the relative contributions of different acetyl-CoA sources to the acetylation of Treg cells.

### HDACs Regulation in Treg Cell by Lactate and Butyrate

In addition to metabolic regulation of HATs, metabolic products such as lactate and butyrate have been identified as inhibitors of HDACs, specifically class I and class II HDACs which are zinc-dependent enzymes. Butyrate, a product of bacterial anaerobic fermentation, enhances histone H3 acetylation in the promoter and CNS regions of the *Foxp3* locus, and eventually facilitates Foxp3 expression in naïve T cell (7, 110). Butyrate can induce the differentiation of peripheral Treg cells (7, 110); in particular, butyrate-induced Treg cells have ability to alleviate chronic intestinal inflammation (7). Lactate, the end product of glucose metabolism, can also inhibit HDACs activity (111), and maintain the suppressive function and proliferation of intratumoral Treg cells (17). In addition, the ketone body β-hydroxybutyrate, closely related to the structure of butyrate, is an endogenous inhibitor of class I HDACs (112). In mouse CD^+^8 memory T cells, β-hydroxybutyrate epigenetically modifies H3K9 of *Foxo1* and *Ppargc1a* (encodes PGC-1α), which upregulate their target gene *Pck1*, thereby directing the carbon flow along the gluconeogenic pathway to glycogen and the pentose phosphate pathway (113). This study reveals β-hydroxybutyrate acts as an unusual metabolite linking epigenetic modification and immune cell metabolism, but this effect has not been studied in Treg cells.

### Sirtuin Regulation in Treg Cells by NAD^+^

Sirtuins (SIRT1-7) belongs to the class III HDAC family, which collectively deacetylates a broad range of histone and non-histone proteins (114). Sirtuins can directly mediate deacetylation of Foxp3 protein, and act as a negative regulator of Treg cell function (115–117). Importantly, sirtuins, specifically SIRT1 and SIRT7 involve in OX40-mediated inhibition of Foxp3 expression and Treg cell induction (118). OX40 upregulates BATF3 and BATF; then they bind to the *Foxp3* locus and recruit the histone deacetylases SIRT1 and SIRT7, which produce a closed chromatin configuration to repress Foxp3 expression (118).

Metabolically, sirtuins are dependent on NAD^+^ availability as a cofactor and are regulated by the NAD^+^/NADH ratio (119). Reduced NAD^+^ levels due to increased glycolytic metabolism have been shown to reduce NAD^+^-dependent HDAC activity (120). A decreased NAD^+^/NADH ratio has been observed in Treg cells that are deficient in complex III, which display a loss of suppressive capacity with a concomitant increase in glycolytic flux (93). These data support the hypothesis that, as a consequence of the increased glycolysis, NAD^+^ exerts a negative influence on Treg cells, at least at some stage. In support of this notion, NAD^+^ directly promotes *in vitro* Treg conversion into Th17 cells (121). However, establishing the mechanistic links between NAD^+^ involved in glycolytic metabolism and variations in sirtuins-regulated acetylation is critical for identifying the role of metabolism-epigenetics in Treg cell development.

### RNA Methylation

RNA methylation forming N6-methyladenosine (m6A) in mRNA has emerged as a new layer of post-transcriptional gene regulation. The deposition of m6A is catalyzed by METTL3–METTL14 complexes, which are SAM-dependent RNA methyltransferase (122). The removal of m6A is achieved by the RNA demethylases FTO and ALKBH5, whose activity depends on α-KG (123, 124). RNA methylation mediated by the RNA methyltransferases METTL3 and METTL14 has been characterized in Treg cells (125–127). METTL14 maintains their differentiation and function (125), whereas METTL3 only affects Treg cell stability but not differentiation (126, 127). Specifically, the depletion of METTL3 in Treg cells leads to increased SOCS mRNA levels, which suppresses the IL-2-STAT5 signaling pathway, resulting in Treg cell dysfunction (127). Moreover, METTL14-deficient Treg cells exhibit decreased RORγt expression, which contributes to their decreased suppressive capacity in colitis (125). It is evident that RNA methylation plays an important role in the gene expression that controls Treg cells. However, it is still unclear how specific RNA methylation sites are differentially regulated by different RNA methyltransferases in Treg cells. In addition, the contribution of metabolite changes to RNA methylation within Treg cells has received relatively little research attention, given the presumed relationship between metabolites and RNA methyltransferases/demethylases. Thus, it is necessary to determine the full scope of involvement of RNA methylation in the differential gene expression of Treg cells and how metabolic alterations are involved in this process.

### O-GlcNAcylation

O-GlcNAcylation is a post-translational modification that reversibly attaches β-N-acetylglucosamine (O-GlcNAc) at the hydroxyl group of serine or threonine residues (128). This process is catalyzed by O-GlcNAc transferase (OGT) and reversed by O-linked GlcNAc hydrolase (OGA) (129). The by-product of the hexosamine biosynthetic pathway, i.e., UDP-GlcNAc, is required as a substrate, which offers a link between metabolic processes and O-GlcNAcylation. In activated T cells, glucose and glutamine are used to fuel O-GlcNAcylation to produce high concentrations of UDP-GlcNAc (130). This process is regulated by c-Myc (130), which maintains Treg cell homeostasis by promoting OXPHOS (131), suggesting that O-GlcNAcylation may therefore influence Treg cell stability. Indeed, a correlation has been revealed between O-GlcNAcylation abundance and Treg cell function, as O-GlcNAcylation can stabilize Foxp3 and activate STAT5 (132). The selective ablation of OGT in Treg cells leads to aggressive autoimmune syndrome in mice as a result of deficiency of Treg cells (132). However, high glucose levels can also enhance cellular O-GlcNAcylation of transcriptional factors such as c-Rel, which negatively regulates Foxp3 expression (133). It seems that the effects of O-GlcNAcylation on Foxp3 are not sufficient to explain all the functions of O-GlcNAcylation in Treg cells. Thus, it is important to deepen our understanding of global O-GlcNAcylation in Treg cells and their association with metabolic reprogramming.

## Conclusion

In summary, there is an intimate link between the metabolism of Treg cells and their epigenetic reprogramming, which in turn plays a coordinated role in their activation, differentiation, and suppressive function. However, as a relatively new area of research, it is not surprising that the studies discussed here have only scratched the surface of the metabolic control of epigenetics in Treg cells. Many questions need to be answered in the future. First, each epigenetic modification can be influenced by metabolites from multiple metabolic pathways, and metabolites from the same pathway can competitively serve as substrates for enzymes or inhibit substrate utilization. To date, studies have focused on only single metabolites. Thus, understanding the relative contributions of metabolites and how the epigenetic modification responds to the status of the entire metabolic network represents important future work. Second, Treg cells are always attuned to local environmental cues that allow the production of intermediates necessary for cell survival or growth. As described above, Treg cells display broad heterogeneity in the metabolism of glucose and lipids within various contextual features. For example, to avoid a negative effect of glycolysis on suppressive function, tumor-infiltrating Treg cells instead upregulate pathways involved in the metabolism of the glycolytic by-product, lactic acid, to maintain suppressive function and proliferation (17). Such metabolism plasticity may be an important consideration in assessing how metabolism reprograms the epigenetic features of Treg cells, especially in non-lymphoid tissues during non-homeostatic states. Finally, no studies reveal only the magnitude of metabolic effects on epigenetic enzymes, independently of other effects, such as transcriptional programs. As a complex relationship certainly exists between metabolism and epigenetics with regards to maintaining Treg cell activation, differentiation, and function, this relationship should be elucidated in future research.

## Author Contributions

JL wrote the article. All authors contributed to the article and approved the submitted version.

## Funding

This work was supported by the National Natural Science Foundation of China (grant number 82073860) and the Young Elite Scientist Sponsorship Program of Henan Association for Science and Technology (grant number 2021HYTP048).

## Conflict of Interest

The authors declare that the research was conducted in the absence of any commercial or financial relationships that could be construed as a potential conflict of interest.

## Publisher’s Note

All claims expressed in this article are solely those of the authors and do not necessarily represent those of their affiliated organizations, or those of the publisher, the editors and the reviewers. Any product that may be evaluated in this article, or claim that may be made by its manufacturer, is not guaranteed or endorsed by the publisher.

## References

[1] Dominguez-Villar M Hafler DA. Regulatory T Cells in Autoimmune Disease. Nat Immunol (2018) 19(7):665–73. 10.1038/s41590-018-0120-4 PMC7882196 29925983

[2] Burzyn D Benoist C Mathis D. Regulatory T Cells in Nonlymphoid Tissues. Nat Immunol (2013) 14(10):1007–13. 10.1038/ni.2683 PMC4708287 24048122

[3] Lu L Barbi J Pan F. The Regulation of Immune Tolerance by FOXP3. Nat Rev Immunol (2017) 17(11):703–17. 10.1038/nri.2017.75 PMC5793224 28757603

[4] Newton R Priyadharshini B Turka LA. Immunometabolism of Regulatory T Cells. Nat Immunol (2016) 17(6):618–25. 10.1038/ni.3466 PMC5006394 27196520

[5] Zhang Q Cao X. Epigenetic Regulation of the Innate Immune Response to Infection. Nat Rev Immunol (2019) 19(7):417–32. 10.1038/s41577-019-0151-6 30918351

[6] Kinnaird A Zhao S Wellen KE Michelakis ED. Metabolic Control of Epigenetics in Cancer. Nat Rev Cancer (2016) 16(11):694–707. 10.1038/nrc.2016.82 27634449

[7] Furusawa Y Obata Y Fukuda S Endo TA Nakato G Takahashi D. Commensal Microbe-Derived Butyrate Induces the Differentiation of Colonic Regulatory T Cells. Nature (2013) 504(7480):446–50. 10.1038/nature12721 24226770

[8] Reid MA Dai Z Locasale JW. The Impact of Cellular Metabolism on Chromatin Dynamics and Epigenetics. Nat Cell Biol (2017) 19(11):1298–306. 10.1038/ncb3629 PMC5886854 29058720

[9] Cavalli G Heard E. Advances in Epigenetics Link Genetics to the Environment and Disease. Nature (2019) 571(7766):489–99. 10.1038/s41586-019-1411-0 31341302

[10] Melkonian EA Schury MP. Biochemistry, Anaerobic Glycolysis. In: StatPearls [Internet]. Treasure Island (FL): StatPearls Publishing (2021). 31536301

[11] Lunt SY Vander Heiden MG. Aerobic Glycolysis: Meeting the Metabolic Requirements of Cell Proliferation. Annu Rev Cell Dev Biol (2011) 27:441–64. 10.1146/annurev-cellbio-092910-154237 21985671

[12] Shi LZ Wang R Huang G Vogel P Neale G Green DR. HIF1alpha-Dependent Glycolytic Pathway Orchestrates a Metabolic Checkpoint for the Differentiation of TH17 and Treg Cells. J Exp Med (2011) 208(7):1367–76. 10.1084/jem.20110278 PMC3135370 21708926

[13] Michalek RD Gerriets VA Jacobs SR Macintyre AN MacIver NJ Mason EF. Cutting Edge: Distinct Glycolytic and Lipid Oxidative Metabolic Programs are Essential for Effector and Regulatory CD4+ T Cell Subsets. J Immunol (2011) 186(6):3299–303. 10.4049/jimmunol.1003613 PMC3198034 21317389

[14] Miska J Lee-Chang C Rashidi A Muroski ME Chang AL Lopez-Rosas A. HIF-1alpha Is a Metabolic Switch Between Glycolytic-Driven Migration and Oxidative Phosphorylation-Driven Immunosuppression of Tregs in Glioblastoma. Cell Rep (2019) 27(1):226–37.e4. 10.1016/j.celrep.2019.03.029 30943404 PMC6461402

[15] Sun IH Oh MH Zhao L Patel CH Arwood ML Xu W. mTOR Complex 1 Signaling Regulates the Generation and Function of Central and Effector Foxp3(+) Regulatory T Cells. J Immunol (2018) 201(2):481–92. 10.4049/jimmunol.1701477 PMC6089237 29884702

[16] Gerriets VA Kishton RJ Johnson MO Cohen S Siska PJ Nichols AG. Foxp3 and Toll-Like Receptor Signaling Balance Treg Cell Anabolic Metabolism for Suppression. Nat Immunol (2016) 17(12):1459–66. 10.1038/ni.3577 PMC5215903 27695003

[17] Watson MJ Vignali PDA Mullett SJ Overacre-Delgoffe AE Peralta RM Grebinoski S. Metabolic Support of Tumour-Infiltrating Regulatory T Cells by Lactic Acid. Nature (2021) 591(7851):645–51. 10.1038/s41586-020-03045-2 PMC7990682 33589820

[18] Procaccini C Carbone F Di Silvestre D Brambilla F De Rosa V Galgani M. The Proteomic Landscape of Human Ex Vivo Regulatory and Conventional T Cells Reveals Specific Metabolic Requirements. Immunity (2016) 44(2):406–21. 10.1016/j.immuni.2016.01.028 PMC4760097 26885861

[19] De Rosa V Galgani M Porcellini A Colamatteo A Santopaolo M Zuchegna C. Glycolysis Controls the Induction of Human Regulatory T Cells by Modulating the Expression of FOXP3 Exon 2 Splicing Variants. Nat Immunol (2015) 16(11):1174–84. 10.1038/ni.3269 PMC4868085 26414764

[20] Lim SA Wei J Nguyen TM Shi H Su W Palacios G. Lipid Signalling Enforces Functional Specialization of Treg Cells in Tumours. Nature (2021) 591(7849):306–11. 10.1038/s41586-021-03235-6 PMC8168716 33627871

[21] Zappasodi R Serganova I Cohen IJ Maeda M Shindo M Senbabaoglu Y. CTLA-4 Blockade Drives Loss of Treg Stability in Glycolysis-Low Tumours. Nature (2021) 591(7851):652–8. 10.1038/s41586-021-03326-4 PMC8057670 33588426

[22] Gerriets VA Kishton RJ Nichols AG Macintyre AN Inoue M Ilkayeva O. Metabolic Programming and PDHK1 Control CD4+ T Cell Subsets and Inflammation. J Clin Invest (2015) 125(1):194–207. 10.1172/JCI76012 25437876 PMC4382238

[23] Angelin A Gil-de-Gomez L Dahiya S Jiao J Guo L Levine MH. Foxp3 Reprograms T Cell Metabolism to Function in Low-Glucose, High-Lactate Environments. Cell Metab (2017) 25(6):1282–93.e7. 10.1016/j.cmet.2016.12.018 28416194 PMC5462872

[24] Sefik E Geva-Zatorsky N Oh S Konnikova L Zemmour D McGuire AM. Individual Intestinal Symbionts Induce a Distinct Population of RORgamma(+) Regulatory T Cells. Science (2015) 349(6251):993–7. 10.1126/science.aaa9420 PMC4700932 26272906

[25] Ohnmacht C Park JH Cording S Wing JB Atarashi K Obata Y. The Microbiota Regulates Type 2 Immunity Through RORgammat(+) T Cells. Science (2015) 349(6251):989–93. 10.1126/science.aac4263 26160380

[26] Song X Sun X Oh SF Wu M Zhang Y Zheng W. Microbial Bile Acid Metabolites Modulate Gut RORgamma(+) Regulatory T Cell Homeostasis. Nature (2020) 577(7790):410–5. 10.1038/s41586-019-1865-0 PMC7274525 31875848

[27] Berod L Friedrich C Nandan A Freitag J Hagemann S Harmrolfs K. De Novo Fatty Acid Synthesis Controls the Fate Between Regulatory T and T Helper 17 Cells. Nat Med (2014) 20(11):1327–33. 10.1038/nm.3704 25282359

[28] Wang H Franco F Tsui YC Xie X Trefny MP Zappasodi R. CD36-Mediated Metabolic Adaptation Supports Regulatory T Cell Survival and Function in Tumors. Nat Immunol (2020) 21(3):298–308. 10.1038/s41590-019-0589-5 32066953 PMC7043937

[29] Chou WC Guo Z Guo H Chen L Zhang G Liang K. AIM2 in Regulatory T Cells Restrains Autoimmune Diseases. Nature (2021) 591(7849):300–5. 10.1038/s41586-021-03231-w PMC8080937 33505023

[30] Pompura SL Wagner A Kitz A LaPerche J Yosef N Dominguez-Villar M. Oleic Acid Restores Suppressive Defects in Tissue-Resident FOXP3 Tregs From Patients With Multiple Sclerosis. J Clin Invest (2021) 131(2):e138519. 10.1172/JCI138519 PMC7810477 33170805

[31] Hosios AM Hecht VC Danai LV Johnson MO Rathmell JC Steinhauser ML. Amino Acids Rather Than Glucose Account for the Majority of Cell Mass in Proliferating Mammalian Cells. Dev Cell (2016) 36(5):540–9. 10.1016/j.devcel.2016.02.012 PMC4766004 26954548

[32] Shi H Chapman NM Wen J Guy C Long L Dhungana Y. Amino Acids License Kinase Mtorc1 Activity and Treg Cell Function via Small G Proteins Rag and Rheb. Immunity (2019) 51(6):1012–27:e7. 10.1016/j.immuni.2019.10.001 PMC6948188 31668641

[33] Ikeda K Kinoshita M Kayama H Nagamori S Kongpracha P Umemoto E. Slc3a2 Mediates Branched-Chain Amino-Acid-Dependent Maintenance of Regulatory T Cells. Cell Rep (2017) 21(7):1824–38. 10.1016/j.celrep.2017.10.082 29141216

[34] Procaccini C Garavelli S Carbone F Di Silvestre D La Rocca C Greco D. Signals of Pseudo-Starvation Unveil the Amino Acid Transporter SLC7A11 as Key Determinant in the Control of Treg Cell Proliferative Potential. Immunity (2021) 54(7):1543–60.e6. 10.1016/j.immuni.2021.04.014 34004141

[35] Fallarino F Grohmann U You S McGrath BC Cavener DR Vacca C. The Combined Effects of Tryptophan Starvation and Tryptophan Catabolites Down-Regulate T Cell Receptor Zeta-Chain and Induce a Regulatory Phenotype in Naive T Cells. J Immunol (2006) 176(11):6752–61. 10.4049/jimmunol.176.11.6752 16709834

[36] Mezrich JD Fechner JH Zhang X Johnson BP Burlingham WJ Bradfield CA. An Interaction Between Kynurenine and the Aryl Hydrocarbon Receptor can Generate Regulatory T Cells. J Immunol (2010) 185(6):3190–8. 10.4049/jimmunol.0903670 PMC2952546 20720200

[37] Nakaya M Xiao Y Zhou X Chang JH Chang M Cheng X. Inflammatory T Cell Responses Rely on Amino Acid Transporter ASCT2 Facilitation of Glutamine Uptake and Mtorc1 Kinase Activation. Immunity (2014) 40(5):692–705. 10.1016/j.immuni.2014.04.007 24792914 PMC4074507

[38] Sinclair LV Rolf J Emslie E Shi YB Taylor PM Cantrell DA. Control of Amino-Acid Transport by Antigen Receptors Coordinates the Metabolic Reprogramming Essential for T Cell Differentiation. Nat Immunol (2013) 14(5):500–8. 10.1038/ni.2556 PMC3672957 23525088

[39] Klysz D Tai X Robert PA Craveiro M Cretenet G Oburoglu L. Glutamine-Dependent Alpha-Ketoglutarate Production Regulates the Balance Between T Helper 1 Cell and Regulatory T Cell Generation. Sci Signaling (2015) 8(396):ra97. 10.1126/scisignal.aab2610 26420908

[40] Metzler B Gfeller P Guinet E. Restricting Glutamine or Glutamine-Dependent Purine and Pyrimidine Syntheses Promotes Human T Cells With High FOXP3 Expression and Regulatory Properties. J Immunol (2016) 196(9):3618–30. 10.4049/jimmunol.1501756 27022197

[41] Kurniawan H Franchina DG Guerra L Bonetti L Baguet LS Grusdat M. Glutathione Restricts Serine Metabolism to Preserve Regulatory T Cell Function. Cell Metab (2020) 31(5):920–36.e7. 10.1016/j.cmet.2020.03.004 32213345 PMC7265172

[42] Kitagawa Y Ohkura N Kidani Y Vandenbon A Hirota K Kawakami R. Guidance of Regulatory T Cell Development by Satb1-Dependent Super-Enhancer Establishment. Nat Immunol (2017) 18(2):173–83. 10.1038/ni.3646 PMC5582804 27992401

[43] Ohkura N Sakaguchi S. Transcriptional and Epigenetic Basis of Treg Cell Development and Function: Its Genetic Anomalies or Variations in Autoimmune Diseases. Cell Res (2020) 30(6):465–74. 10.1038/s41422-020-0324-7 PMC7264322 32367041

[44] Delacher M Imbusch CD Hotz-Wagenblatt A Mallm JP Bauer K Simon M. Precursors for Nonlymphoid-Tissue Treg Cells Reside in Secondary Lymphoid Organs and Are Programmed by the Transcription Factor BATF. Immunity (2020) 52(2):295–312.e11. 10.1016/j.immuni.2019.12.002 31924477 PMC7026712

[45] Delacher M Simon M Sanderink L Hotz-Wagenblatt A Wuttke M Schambeck K. Single-Cell Chromatin Accessibility Landscape Identifies Tissue Repair Program in Human Regulatory T Cells. Immunity (2021) 54(4):702–20.e17. 10.1016/j.immuni.2021.03.007 33789089 PMC8050210

[46] Delacher M Imbusch CD Weichenhan D Breiling A Hotz-Wagenblatt A Träger U. Genome-Wide DNA-Methylation Landscape Defines Specialization of Regulatory T Cells in Tissues. Nat Immunol (2017) 18(10):1160–72. 10.1038/ni.3799 PMC5912503 28783152

[47] Ohkura N Hamaguchi M Morikawa H Sugimura K Tanaka A Ito Y. T Cell Receptor Stimulation-Induced Epigenetic Changes and Foxp3 Expression are Independent and Complementary Events Required for Treg Cell Development. Immunity (2012) 37(5):785–99. 10.1016/j.immuni.2012.09.010 23123060

[48] Morales-Nebreda L McLafferty FS Singer BD. DNA Methylation as a Transcriptional Regulator of the Immune System. Transl Res (2019) 204:1–18. 10.1016/j.trsl.2018.08.001 30170004 PMC6331288

[49] Jones PA. Functions of DNA Methylation: Islands, Start Sites, Gene Bodies and Beyond. Nat Rev Genet (2012) 13(7):484–92. 10.1038/nrg3230 22641018

[50] Bannister AJ Kouzarides T. Regulation of Chromatin by Histone Modifications. Cell Res (2011) 21(3):381–95. 10.1038/cr.2011.22 PMC3193420 21321607

[51] Floess S Freyer J Siewert C Baron U Olek S Polansky J. Epigenetic Control of the Foxp3 Locus in Regulatory T Cells. PloS Biol (2007) 5(2):e38. 10.1371/journal.pbio.0050038 17298177 PMC1783672

[52] Kawakami R Kitagawa Y Chen KY Arai M Ohara D Nakamura Y. Distinct Foxp3 Enhancer Elements Coordinate Development, Maintenance, and Function of Regulatory T Cells. Immunity (2021) 54(5):947–61.e8. 10.1016/j.immuni.2021.04.005 33930308

[53] Zheng Y Josefowicz S Chaudhry A Peng XP Forbush K Rudensky AY. Role of Conserved non-Coding DNA Elements in the Foxp3 Gene in Regulatory T-Cell Fate. Nature (2010) 463(7282):808–12. 10.1038/nature08750 PMC2884187 20072126

[54] Polansky JK Kretschmer K Freyer J Floess S Garbe A Baron U. DNA Methylation Controls Foxp3 Gene Expression. Eur J Immunol (2008) 38(6):1654–63. 10.1002/eji.200838105 18493985

[55] Mikami N Kawakami R Chen KY Sugimoto A Ohkura N Sakaguchi S. Epigenetic Conversion of Conventional T Cells Into Regulatory T Cells by CD28 Signal Deprivation. Proc Natl Acad Sci USA (2020) 117(22):12258–68. 10.1073/pnas.1922600117 PMC7275710 32414925

[56] Helmin KA Morales-Nebreda L Acosta MAT Anekalla KR Chen SY Abdala-Valencia H. Maintenance DNA Methylation Is Essential for Regulatory T Cell Development and Stability of Suppressive Function. J Clin Invest (2020) 130(12):6571–87. 10.1172/JCI137712 PMC7710299 32897881

[57] Obata Y Furusawa Y Endo TA Sharif J Takahashi D Atarashi K. The Epigenetic Regulator Uhrf1 Facilitates the Proliferation and Maturation of Colonic Regulatory T Cells. Nat Immunol (2014) 15(6):571–9. 10.1038/ni.2886 24777532

[58] Kouzarides T. Chromatin Modifications and Their Function. Cell (2007) 128(4):693–705. 10.1016/j.cell.2007.02.005 17320507

[59] Wei G Wei L Zhu J Zang C Hu-Li J Yao Z. Global Mapping of H3K4me3 and H3K27me3 Reveals Specificity and Plasticity in Lineage Fate Determination of Differentiating CD4+ T Cells. Immunity (2009) 30(1):155–67. 10.1016/j.immuni.2008.12.009 PMC2722509 19144320

[60] He H Ni B Tian Y Tian Z Chen Y Liu Z. Histone Methylation Mediates Plasticity of Human FOXP3(+) Regulatory T Cells by Modulating Signature Gene Expressions. Immunology (2014) 141(3):362–76. 10.1111/imm.12198 PMC3930375 24152290

[61] Morikawa H Ohkura N Vandenbon A Itoh M Nagao-Sato S Kawaji H. Differential Roles of Epigenetic Changes and Foxp3 Expression in Regulatory T Cell-Specific Transcriptional Regulation. Proc Natl Acad Sci USA (2014) 111(14):5289–94. 10.1073/pnas.1312717110 PMC3986152 24706905

[62] Arvey A van der Veeken J Samstein RM Feng Y Stamatoyannopoulos JA Rudensky AY. Inflammation-Induced Repression of Chromatin Bound by the Transcription Factor Foxp3 in Regulatory T Cells. Nat Immunol (2014) 15(6):580–7. 10.1038/ni.2868 PMC4112080 24728351

[63] Mijnheer G Lutter L Mokry M van der Wal M Scholman R Fleskens V. Conserved Human Effector Treg Cell Transcriptomic and Epigenetic Signature in Arthritic Joint Inflammation. Nat Commun (2021) 12(1):2710. 10.1038/s41467-021-22975-7 33976194 PMC8113485

[64] Tao R de Zoeten EF Ozkaynak E Chen C Wang L Porrett PM. Deacetylase Inhibition Promotes the Generation and Function of Regulatory T Cells. Nat Med (2007) 13(11):1299–307. 10.1038/nm1652 17922010

[65] Ohkura N Yasumizu Y Kitagawa Y Tanaka A Nakamura Y Motooka D. Regulatory T Cell-Specific Epigenomic Region Variants Are a Key Determinant of Susceptibility to Common Autoimmune Diseases. Immunity (2020) 52(6):1119–32.e4. 10.1016/j.immuni.2020.04.006 32362325

[66] Feng Y van der Veeken J Shugay M Putintseva EV Osmanbeyoglu HU Dikiy S. A Mechanism for Expansion of Regulatory T-Cell Repertoire and its Role in Self-Tolerance. Nature (2015) 528(7580):132–6. 10.1038/nature16141 PMC4862833 26605529

[67] Arvey A van der Veeken J Plitas G Rich SS Concannon P Rudensky AY. Genetic and Epigenetic Variation in the Lineage Specification of Regulatory T Cells. eLife (2015) 4:e07571. 10.7554/eLife.07571 26510014 PMC4623597

[68] Buenrostro JD Giresi PG Zaba LC Chang HY Greenleaf WJ. Transposition of Native Chromatin for Fast and Sensitive Epigenomic Profiling of Open Chromatin, DNA-Binding Proteins and Nucleosome Position. Nat Methods (2013) 10(12):1213–8. 10.1038/nmeth.2688 PMC3959825 24097267

[69] Vasanthakumar A Chisanga D Blume J Gloury R Britt K Henstridge DC. Sex-Specific Adipose Tissue Imprinting of Regulatory T Cells. Nature (2020) 579(7800):581–5. 10.1038/s41586-020-2040-3 PMC7241647 32103173

[70] Hayatsu N Miyao T Tachibana M Murakami R Kimura A Kato T. Analyses of a Mutant Foxp3 Allele Reveal BATF as a Critical Transcription Factor in the Differentiation and Accumulation of Tissue Regulatory T Cells. Immunity (2017) 47(2):268–83.e9. 10.1016/j.immuni.2017.07.008 28778586

[71] Singer BD Mock JR Aggarwal NR Garibaldi BT Sidhaye VK Florez MA. Regulatory T Cell DNA Methyltransferase Inhibition Accelerates Resolution of Lung Inflammation. Am J Respir Cell Mol Biol (2015) 52(5):641–52. 10.1165/rcmb.2014-0327OC PMC4491142 25295995

[72] Lu CH Wu CJ Chan CC Nguyen DT Lin KR Lin SJ. DNA Methyltransferase Inhibitor Promotes Human CD4(+)CD25(h)FOXP3(+) Regulatory T Lymphocyte Induction Under Suboptimal TCR Stimulation. Front Immunol (2016) 7:488. 10.3389/fimmu.2016.00488 27877174 PMC5099256

[73] Chan MW Chang CB Tung CH Sun J Suen JL Wu SF. Low-Dose 5-Aza-2’-Deoxycytidine Pretreatment Inhibits Experimental Autoimmune Encephalomyelitis by Induction of Regulatory T Cells. Mol Med (2014) 20:248–56. 10.2119/molmed.2013.00159 PMC4107100 24869907

[74] Kim HP Leonard WJ. CREB/ATF-Dependent T Cell Receptor-Induced FoxP3 Gene Expression: A Role for DNA Methylation. J Exp Med (2007) 204(7):1543–51. 10.1084/jem.20070109 PMC2118651 17591856

[75] Ducker GS Rabinowitz JD. One-Carbon Metabolism in Health and Disease. Cell Metab (2017) 25(1):27–42. 10.1016/j.cmet.2016.08.009 27641100 PMC5353360

[76] Locasale JW. Serine, Glycine and One-Carbon Units: Cancer Metabolism in Full Circle. Nat Rev Cancer (2013) 13(8):572–83. 10.1038/nrc3557 PMC3806315 23822983

[77] Yang M Vousden KH. Serine and One-Carbon Metabolism in Cancer. Nat Rev Cancer (2016) 16(10):650–62. 10.1038/nrc.2016.81 27634448

[78] Mentch SJ Mehrmohamadi M Huang L Liu X Gupta D Mattocks D. Histone Methylation Dynamics and Gene Regulation Occur Through the Sensing of One-Carbon Metabolism. Cell Metab (2015) 22(5):861–73. 10.1016/j.cmet.2015.08.024 PMC4635069 26411344

[79] Pfalzer AC Choi SW Tammen SA Park LK Bottiglieri T Parnell LD. S-Adenosylmethionine Mediates Inhibition of Inflammatory Response and Changes in DNA Methylation in Human Macrophages. Physiol Genomics (2014) 46(17):617–23. 10.1152/physiolgenomics.00056.2014 25180283

[80] Moore JR Hubler SL Nelson CD Nashold FE Spanier JA Hayes CE. 1,25-Dihydroxyvitamin D3 Increases the Methionine Cycle, CD4(+) T Cell DNA Methylation and Helios(+)Foxp3(+) T Regulatory Cells to Reverse Autoimmune Neurodegenerative Disease. J Neuroimmunol (2018) 324:100–14. 10.1016/j.jneuroim.2018.09.008 30267995

[81] Lu L Lan Q Li Z Zhou X Gu J Li Q. Critical Role of All-Trans Retinoic Acid in Stabilizing Human Natural Regulatory T Cells Under Inflammatory Conditions. Proc Natl Acad Sci USA (2014) 111(33):E3432–40. 10.1073/pnas.1408780111 PMC4143025 25099355

[82] Golovina TN Mikheeva T Brusko TM Blazar BR Bluestone JA Riley JL. Retinoic Acid and Rapamycin Differentially Affect and Synergistically Promote the Ex Vivo Expansion of Natural Human T Regulatory Cells. PloS One (2011) 6(1):e15868. 10.1371/journal.pone.0015868 21253593 PMC3017077

[83] Yang R Qu C Zhou Y Konkel JE Shi S Liu Y. Hydrogen Sulfide Promotes Tet1- and Tet2-Mediated Foxp3 Demethylation to Drive Regulatory T Cell Differentiation and Maintain Immune Homeostasis. Immunity (2015) 43(2):251–63. 10.1016/j.immuni.2015.07.017 PMC4731232 26275994

[84] Li Q Zou J Wang M Ding X Chepelev I Zhou X. Critical Role of Histone Demethylase Jmjd3 in the Regulation of CD4+ T-Cell Differentiation. Nat Commun (2014) 5:5780. 10.1038/ncomms6780 25531312 PMC4274750

[85] Chisolm DA Weinmann AS. Connections Between Metabolism and Epigenetics in Programming Cellular Differentiation. Annu Rev Immunol (2018) 36:221–46. 10.1146/annurev-immunol-042617-053127 29328786

[86] Xiao M Yang H Xu W Ma S Lin H Zhu H. Inhibition of Alpha-KG-Dependent Histone and DNA Demethylases by Fumarate and Succinate That are Accumulated in Mutations of FH and SDH Tumor Suppressors. Genes Dev (2012) 26(12):1326–38. 10.1101/gad.191056.112 PMC3387660 22677546

[87] Martinez-Reyes I Chandel NS. Mitochondrial TCA Cycle Metabolites Control Physiology and Disease. Nat Commun (2020) 11(1):102. 10.1038/s41467-019-13668-3 31900386 PMC6941980

[88] Carey BW Finley LW Cross JR Allis CD Thompson CB. Intracellular Alpha-Ketoglutarate Maintains the Pluripotency of Embryonic Stem Cells. Nature (2015) 518(7539):413–6. 10.1038/nature13981 PMC4336218 25487152

[89] Xu T Stewart KM Wang X Liu K Xie M Ryu JK. Metabolic Control of TH17 and Induced Treg Cell Balance by an Epigenetic Mechanism. Nature (2017) 548(7666):228–33. 10.1038/nature23475 PMC6701955 28783731

[90] Picca A Lezza AM. Regulation of Mitochondrial Biogenesis Through TFAM-Mitochondrial DNA Interactions: Useful Insights From Aging and Calorie Restriction Studies. Mitochondrion (2015) 25:67–75. 10.1016/j.mito.2015.10.001 26437364

[91] Fu Z Ye J Dean JW Bostick JW Weinberg SE Xiong L. Requirement of Mitochondrial Transcription Factor A in Tissue-Resident Regulatory T Cell Maintenance and Function. Cell Rep (2019) 28(1):159–71.e4. 10.1016/j.celrep.2019.06.024 31269437 PMC6679941

[92] Liu X Zhang Y Ni M Cao H Signer RAJ Li D. Regulation of Mitochondrial Biogenesis in Erythropoiesis by mTORC1-Mediated Protein Translation. Nat Cell Biol (2017) 19(6):626–38. 10.1038/ncb3527 PMC5771482 28504707

[93] Weinberg SE Singer BD Steinert EM Martinez CA Mehta MM Martinez-Reyes I. Mitochondrial Complex III is Essential for Suppressive Function of Regulatory T Cells. Nature (2019) 565(7740):495–9. 10.1038/s41586-018-0846-z PMC6345596 30626970

[94] Wapenaar H Dekker FJ. Histone Acetyltransferases: Challenges in Targeting Bi-Substrate Enzymes. Clin Epigenet (2016) 8:59. 10.1186/s13148-016-0225-2 PMC4881052 27231488

[95] Xiao Y Nagai Y Deng G Ohtani T Zhu Z Zhou Z. Dynamic Interactions Between TIP60 and P300 Regulate FOXP3 Function Through a Structural Switch Defined by a Single Lysine on TIP60. Cell Rep (2014) 7(5):1471–80. 10.1016/j.celrep.2014.04.021 PMC4064594 24835996

[96] Wang L Kumar S Dahiya S Wang F Wu J Newick K. Ubiquitin-Specific Protease-7 Inhibition Impairs Tip60-Dependent Foxp3+ T-Regulatory Cell Function and Promotes Antitumor Immunity. EBioMedicine (2016) 13:99–112. 10.1016/j.ebiom.2016.10.018 27769803 PMC5264272

[97] Liu Y Wang L Predina J Han R Beier UH Wang LC. Inhibition of P300 Impairs Foxp3(+) T Regulatory Cell Function and Promotes Antitumor Immunity. Nat Med (2013) 19(9):1173–7. 10.1038/nm.3286 PMC3793393 23955711

[98] Castillo J Wu E Lowe C Srinivasan S McCord R Wagle MC. CBP/p300 Drives the Differentiation of Regulatory T Cells Through Transcriptional and Non-Transcriptional Mechanisms. Cancer Res (2019) 79(15):3916–27. 10.1158/0008-5472.CAN-18-3622 31182547

[99] Meier JL. Metabolic Mechanisms of Epigenetic Regulation. ACS Chem Biol (2013) 8(12):2607–21. 10.1021/cb400689r PMC3918459 24228614

[100] Montgomery DC Sorum AW Guasch L Nicklaus MC Meier JL. Metabolic Regulation of Histone Acetyltransferases by Endogenous Acyl-CoA Cofactors. Chem Biol (2015) 22(8):1030–9. 10.1016/j.chembiol.2015.06.015 PMC4546520 26190825

[101] Sabari BR Tang Z Huang H Yong-Gonzalez V Molina H Kong HE. Intracellular Crotonyl-CoA Stimulates Transcription Through P300-Catalyzed Histone Crotonylation. Mol Cell (2015) 58(2):203–15. 10.1016/j.molcel.2015.02.029 PMC4501262 25818647

[102] Cai L Sutter BM Li B Tu BP. Acetyl-CoA Induces Cell Growth and Proliferation by Promoting the Acetylation of Histones at Growth Genes. Mol Cell (2011) 42(4):426–37. 10.1016/j.molcel.2011.05.004 PMC3109073 21596309

[103] Wellen KE Hatzivassiliou G Sachdeva UM Bui TV Cross JR Thompson CB. ATP-Citrate Lyase Links Cellular Metabolism to Histone Acetylation. Science (2009) 324(5930):1076–80. 10.1126/science.1164097 PMC2746744 19461003

[104] Takahashi H McCaffery JM Irizarry RA Boeke JD. Nucleocytosolic Acetyl-Coenzyme a Synthetase is Required for Histone Acetylation and Global Transcription. Mol Cell (2006) 23(2):207–17. 10.1016/j.molcel.2006.05.040 16857587

[105] McDonnell E Crown SB Fox DB Kitir B Ilkayeva OR Olsen CA. Lipids Reprogram Metabolism to Become a Major Carbon Source for Histone Acetylation. Cell Rep (2016) 17(6):1463–72. 10.1016/j.celrep.2016.10.012 PMC5123807 27806287

[106] Cluntun AA Huang H Dai L Liu X Zhao Y Locasale JW. The Rate of Glycolysis Quantitatively Mediates Specific Histone Acetylation Sites. Cancer Metab (2015) 3:10. 10.1186/s40170-015-0135-3 26401273 PMC4579576

[107] Pietrocola F Galluzzi L Bravo-San Pedro JM Madeo F Kroemer G. Acetyl Coenzyme A: A Central Metabolite and Second Messenger. Cell Metab (2015) 21(6):805–21. 10.1016/j.cmet.2015.05.014 26039447

[108] Peng M Yin N Chhangawala S Xu K Leslie CS Li MO. Aerobic Glycolysis Promotes T Helper 1 Cell Differentiation Through an Epigenetic Mechanism. Science (2016) 354(6311):481–4. 10.1126/science.aaf6284 PMC5539971 27708054

[109] Hang S Paik D Yao L Kim E Trinath J Lu J. Bile Acid Metabolites Control TH17 and Treg Cell Differentiation. Nature (2019) 576(7785):143–8. 10.1038/s41586-019-1785-z PMC6949019 31776512

[110] Arpaia N Campbell C Fan X Dikiy S van der Veeken J deRoos P. Metabolites Produced by Commensal Bacteria Promote Peripheral Regulatory T-Cell Generation. Nature (2013) 504(7480):451–5. 10.1038/nature12726 PMC3869884 24226773

[111] Latham T Mackay L Sproul D Karim M Culley J Harrison DJ. Lactate, a Product of Glycolytic Metabolism, Inhibits Histone Deacetylase Activity and Promotes Changes in Gene Expression. Nucleic Acids Res (2012) 40(11):4794–803. 10.1093/nar/gks066 PMC3367171 22323521

[112] Newman JC Verdin E. Beta-Hydroxybutyrate: Much More Than a Metabolite. Diabetes Res Clin Pract (2014) 106(2):173–81. 10.1016/j.diabres.2014.08.009 PMC4414487 25193333

[113] Zhang H Tang K Ma J Zhou L Liu J Zeng L. Ketogenesis-Generated Beta-Hydroxybutyrate is an Epigenetic Regulator of CD8(+) T-Cell Memory Development. Nat Cell Biol (2020) 22(1):18–25. 10.1038/s41556-019-0440-0 31871320

[114] Sebastian C Satterstrom FK Haigis MC Mostoslavsky R. From Sirtuin Biology to Human Diseases: An Update. J Biol Chem (2012) 287(51):42444–52. 10.1074/jbc.R112.402768 PMC3522245 23086954

[115] van Loosdregt J Brunen D Fleskens V Pals CE Lam EW Coffer PJ. Rapid Temporal Control of Foxp3 Protein Degradation by Sirtuin-1. PloS One (2011) 6(4):e19047. 10.1371/journal.pone.0019047 21533107 PMC3080399

[116] Beier UH Wang L Bhatti TR Liu Y Han R Ge G. Sirtuin-1 Targeting Promotes Foxp3+ T-Regulatory Cell Function and Prolongs Allograft Survival. Mol Cell Biol (2011) 31(5):1022–9. 10.1128/MCB.01206-10 PMC3067815 21199917

[117] Marcel N Perumalsamy LR Shukla SK Sarin A. The Lysine Deacetylase Sirtuin 1 Modulates the Localization and Function of the Notch1 Receptor in Regulatory T Cells. Sci Signaling (2017) 10(473):eaah4679. 10.1126/scisignal.aah4679 28377411

[118] Zhang X Xiao X Lan P Li J Dou Y Chen W. OX40 Costimulation Inhibits Foxp3 Expression and Treg Induction via BATF3-Dependent and Independent Mechanisms. Cell Rep (2018) 24(3):607–18. 10.1016/j.celrep.2018.06.052 PMC6095196 30021159

[119] Chalkiadaki A Guarente L. Sirtuins Mediate Mammalian Metabolic Responses to Nutrient Availability. Nat Rev Endocrinol (2012) 8(5):287–96. 10.1038/nrendo.2011.225 22249520

[120] Ryall JG Dell’Orso S Derfoul A Juan A Zare H Feng X. The NAD(+)-Dependent SIRT1 Deacetylase Translates a Metabolic Switch Into Regulatory Epigenetics in Skeletal Muscle Stem Cells. Cell Stem Cell (2015) 16(2):171–83. 10.1016/j.stem.2014.12.004 PMC4320668 25600643

[121] Elkhal A Rodriguez Cetina Biefer H Heinbokel T Uehara H Quante M Seyda M. NAD(+) Regulates Treg Cell Fate and Promotes Allograft Survival via a Systemic IL-10 Production That is CD4(+) CD25(+) Foxp3(+) T Cells Independent. Sci Rep (2016) 6:22325. 10.1038/srep22325 26928119 PMC4772111

[122] Pendleton KE Chen B Liu K Hunter OV Xie Y Tu BP. The U6 snRNA M(6)A Methyltransferase METTL16 Regulates SAM Synthetase Intron Retention. Cell (2017) 169 824-835(5):e14. 10.1016/j.cell.2017.05.003 PMC5502809 28525753

[123] Aik W Demetriades M Hamdan MK Bagg EA Yeoh KK Lejeune C. Structural Basis for Inhibition of the Fat Mass and Obesity Associated Protein (FTO). J Med Chem (2013) 56(9):3680–8. 10.1021/jm400193d 23547775

[124] Gerken T Girard CA Tung YC Webby CJ Saudek V Hewitson KS. The Obesity-Associated FTO Gene Encodes a 2-Oxoglutarate-Dependent Nucleic Acid Demethylase. Science (2007) 318(5855):1469–72. 10.1126/science.1151710 PMC2668859 17991826

[125] Lu TX Zheng Z Zhang L Sun HL Bissonnette M Huang H. A New Model of Spontaneous Colitis in Mice Induced by Deletion of an RNA M(6)A Methyltransferase Component METTL14 in T Cells. Cell Mol Gastroenterol Hepatol (2020) 10(4):747–61. 10.1016/j.jcmgh.2020.07.001 PMC7498954 32634481

[126] Li HB Tong J Zhu S Batista PJ Duffy EE Zhao J. M(6)A mRNA Methylation Controls T Cell Homeostasis by Targeting the IL-7/STAT5/SOCS Pathways. Nature (2017) 548(7667):338–42. 10.1038/nature23450 PMC5729908 28792938

[127] Tong J Cao G Zhang T Sefik E Amezcua Vesely MC Broughton JP. M(6)A mRNA Methylation Sustains Treg Suppressive Functions. Cell Res (2018) 28(2):253–6. 10.1038/cr.2018.7 PMC5799823 29303144

[128] Yang X Qian K. Protein O-GlcNAcylation: Emerging Mechanisms and Functions. Nature Reviews. Mol Cell Biol (2017) 18(7):452–65. 10.1038/nrm.2017.22 PMC5667541 28488703

[129] Ma J Wu C Hart GW. Analytical and Biochemical Perspectives of Protein O-GlcNAcylation. Chem Rev (2021) 121(3):1513–81. 10.1021/acs.chemrev.0c00884 33416322

[130] Swamy M Pathak S Grzes KM Damerow S Sinclair LV van Aalten DM. Glucose and Glutamine Fuel Protein O-GlcNAcylation to Control T Cell Self-Renewal and Malignancy. Nat Immunol (2016) 17(6):712–20. 10.1038/ni.3439 PMC4900450 27111141

[131] Saravia J Zeng H Dhungana Y Bastardo Blanco D Nguyen TM Chapman NM. Homeostasis and Transitional Activation of Regulatory T Cells Require C-Myc. Sci Adv (2020) 6(1):eaaw6443. 10.1126/sciadv.aaw6443 31911938 PMC6938709

[132] Liu B Salgado OC Singh S Hippen KL Maynard JC Burlingame AL. The Lineage Stability and Suppressive Program of Regulatory T Cells Require Protein O-GlcNAcylation. Nat Commun (2019) 10(1):354. 10.1038/s41467-019-08300-3 30664665 PMC6341091

[133] de Jesus TJ Tomalka JA Centore JT Staback Rodriguez FD Agarwal RA Liu AR. Negative Regulation of FOXP3 Expression by C-Rel O-GlcNAcylation. Glycobiology (2021). 10.1093/glycob/cwab001 [Epub ahead of print] PMC8351495 33442719

